# Nutritional supplements modulate fluorescent protein-bound advanced glycation endproducts and digestive enzymes related to type 2 diabetes mellitus

**DOI:** 10.1186/s12906-016-1329-0

**Published:** 2016-09-01

**Authors:** Emily R. Koch, Permal Deo

**Affiliations:** School of Pharmacy and Medical Sciences, University of the South Australia, Adelaide, SA 5001 Australia

**Keywords:** Liquid nutritional supplements, Antiglycation, α-amylase, α-glucosidase, Phenolic, Flavonoids, Antioxidant activities

## Abstract

**Background:**

Chronic hyperglycemia enhances the formation of advanced glycation endproducts (AGEs) and reactive oxygen species (ROS), contributing to diabetic complications. Thus, controlling blood glucose levels, inhibiting the formation of AGEs and reducing ROS are key therapeutic targets in early stage type 2 diabetes.

**Methods:**

The inhibitory effects of seven commercial liquid nutritional supplements against carbohydrate hydrolysing enzymes, α-amylase and α-glucosidase, was determined by dinitrosalicylic (DNS) reagent and *p*-nitrophenyl-α-D-glucopyranoside solution, respectively. Antiglycation activity was determined using the formation of fluorescent protein-bound AGEs. Total phenolic and flavonoid content and antioxidant properties (1,1-diphenyl-2-picrylhydrazyl antioxidant activity (DPPH) and ferric reducing antioxidant power (FRAP)) were determined for correlation among these components and inhibitory activities.

**Results:**

Samoan noni juice showed the greatest inhibitory effects against α-amylase, whereas chlorophyll extracts showed the greatest inhibitory effect against α-glucosidase. Inhibition of α-glucosidase correlated with TFC (r^2^ = 0.766; *p* < 0.01) and FRAP (r^2^ = 0.750; *p* < 0.01) whereas no correlation was observed for α-amylase inhibition. All supplements inhibited fluorescent protein-bound AGEs, with the greatest effect exerted by Olive Leaf Extract, Blood Sugar Support (IC_50_ = 0.5 mg/ml). The IC_50_ values negatively correlated with TPC (r^2^ = −0.707; *p* < 0.001) and DPPH scavenging activities (r^2^ = 0.515; *p* < 0.05).

**Conclusion:**

The findings of this study highlight the potential of liquid nutritional supplements in managing and treating type 2 diabetes mellitus.

## Background

The early stages of type 2 diabetes mellitus (T2DM) is characterised by postprandial hyperglycemia due to increased breakdown of starch by α-amylase, absorption of glucose by α-glycosidase, insulin resistance and defects with insulin secretion from beta cells of the pancreas [1, 2]. If unmanaged, chronic hyperglycemia can promote protein glycation and the formation of advanced glycation endproducts (AGEs). Protein glycation is initiated by the nucleophilic attack from a carbonyl group of a reducing sugar to a free amine group present on proteins, lipids and nucleic acids. This forms a freely reversible Schiff base and rearranges to form a more stable and irreversible ketoamine or Amadori product that leads to the formation of AGEs [3, 4]. Elevated levels of AGEs in the body contribute to diabetic complications and age related disease by directly causing the cross-linking of long lived proteins such as collagen, leading to vascular stiffness and affecting vascular structure and function. AGEs can also interact with receptors, such as the receptor for AGEs, to induce intracellular signalling that amplifies oxidative stress and transcription of pro-inflammatory markers [1, 4]. Chronic hyperglycemia also promotes the formation of free radicals, that further increases oxidative stress and the formation of reactive oxygen species (ROS), which in turn accelerates AGE formation [3, 5].

Improving glycaemic control and preventing postprandial hyperglycemia is one of the main targets for the treatment and management of the early stage of T2DM. Salivary and pancreatic α-amylase and intestinal α-glucosidase are responsible for the digestion and absorption of glucose from the diet. By inhibiting either of these enzymes the amount of glucose absorbed into the blood following a meal can be reduced, thus preventing the postprandial surge in glucose and maintaining glucose homeostasis [6, 7]. Acarbose, a synthetic drug and a known α-glucosidase and α-amylase inhibitor, is currently prescribed to type 2 diabetic patients. Whilst acarbose is effective in controlling blood glucose levels, its adverse side effects includes gastrointestinal disturbances such as diarrhoea, flatulence and abdominal distension [8]. Recently, there has been an increasing search for natural alternatives including neutraceutical formulations for their potent antioxidant activities and health related benefits. Polyphenols, abundant in plant based foods and beverages, are well known for their potent antioxidant effects [9, 10]. Recent studies have also shown polyphenols from plant extracts to be effective inhibitors of α-amylase [11, 12], α-glucosidase [11, 12] and protein glycation based on in vitro and in vivo studies [5, 13, 14].

Liquid nutritional supplements, a class of neutraceuticals including herbal extracts and plant-based extracts, claim to contain high levels of polyphenols and may be a suitable and cost-effective alternative to synthetic drugs. Very little scientific evidence exists concerning dietary supplements and their health benefits, and none to our knowledge on commercial liquid nutritional supplements on their potential role in the management of hyperglycemia and T2DM. Thus, this study was aimed to investigate the inhibitory effect of commonly consumed liquid nutritional supplements against α-amylase, α-glucosidase, and AGE formation using in vitro model. Total phenolic and flavonoid content, and antioxidant properties were evaluated for correlation among these components and inhibitory activities.

## Methods

### Reagents

Chemicals and solvents were purchased from Sigma-Aldrich (Sydney, Australia) unless otherwise specified. All chemicals were either analytical or HPLC grade.

### Samples

Liquid nutritional supplements, purchased from local retailers and online included: Acai Power, Acai Juice Blend (APNG, containing acai concentrate, pear and cherry juice); Chlorophyll, Detox Health Drink (CHC, containing chlorophyll powder); Olive Leaf Extract, Natural High Strength (OLE01, containing olive leaf extracts with 4.4 mg/ mL oleuropein); Olive Leaf Extract, High Strength, (OLE02, containing olive leaf extracts with 4.4 mg/mL oleuropein and mixed berries); Olive Leaf Extract, Blood Sugar Support (OLE03, containing olive leaf extracts with 4.4 mg/mL oleuropein and 0.25 mg/mL hydroxytyrosol); Noni Juice, Samoan (NJ01, containing 100 % noni fruit extracts); Fijian Noni (NJ02, containing 100 % Fijian noni extracts).

### Inhibition of α-amylase assay

The α-amylase inhibitory action was determined as described previously with some modifications [15]. Samples (125μL, 1 mg/mL) were incubated with porcine pancreatic α-amylase solution (125μL, 0.5 mg/mL in 0.1 M phosphate buffer saline (pH 6.9)), at 37 °C for 10 min. After pre-incubation starch solution (125μL, 1 %) was added at timed intervals (~30μL every 10 s) and the reaction mixture was further incubated at 37 °C for 30 min. The reaction was stopped by adding DNS reagent (250μL, containing 1 % 3,5-dinitrosalicylic acid, 0.2 % phenol, 0.5 % sodium sulphite and 1 % sodium hydroxide) and heating in a boiling water bath (100 °C) for 10 min. After boiling the colour was stabilized with the addition of potassium sodium tartarate solution (250μL, 40 %). The mixture was cooled to room temperature and the absorbance was measured at 540 nm. Acarbose was used in the assay as positive control. Inhibitory activity was expressed as % inhibition = ((A_control_ – A_sample_)/A_control_) × 100.

### Inhibition of α-glucosidase assay

The α-glucosidase inhibitory action was determined as described previously with some modifications [15]. Diluted samples (50μL, 1 mg/mL) and α-glucosidase solution (100μL, 0.2 units/mL in 0.1 M phosphate buffer (pH 6.9)), were mixed in a 96 well plate and incubated at 37 °C for 30 min. After pre-incubation, *p*-nitrophenyl-α-D-glucopyranoside solution (50μL, 5 mM in 0.1 M phosphate buffer (pH 6.9)), was added to the reaction mixture and incubated at 37 °C for 30 min. Sodium carbonate solution (60 μL, 0.1 M) was added and the reaction mixture was further incubated at 37 °C for 20 min. Absorbance was measured at 450 nm. Acarbose was used as a positive control. Inhibitory activity of α-glucosidase was expressed as the % inhibition = (A_control_ – A_sample_)/A_control_) × 100.

### Fluorescence AGE formation assay

Fluorescent protein-bound AGEs were measured according to a previous method with modification [5]. Briefly, 1000μL of bovine serum albumin (BSA, 10 mg/mL final concentration), prepared with 0.2 M phosphate buffer (pH 7.4), was incubated with nutritional supplements (200μL, 0.5-5 mg/mL final concentration) at room temperature for 30 mins. After pre-incubation, glucose (800μL, 100 mM final concentration) was added to each reaction vessel, flushed with nitrogen and incubated at 37 °C for 3 weeks. Aminoguanidine (30μM final concentration) was included as a positive control. All reaction vessels contained 0.02 % sodium azide (final concentration) to prevent any microbial growth. After 3 weeks incubation, protein was isolated by slowly adding trichloroacetic acid (400μL, 20 %) to 500μL of glycated sample. The mixture was kept on ice for 10 min before centrifugation (10000 × g) for 10 mins. Pellet was dissolved in alkaline phosphate buffer (1000μL, 0.2 M, pH 10) and fluorescence was read at excitation and emission wavelengths of 370 nm Ex and 440 nm, respectively. Percent inhibition was determined and IC_50_ values were calculated as % inhibition = ((F_negative control_ – F_sample_)/F_negative control_) × 100.

### Total phenolic and total flavonoid content

Total phenolic content was determined using Folin–Ciocalteu assay and total flavonoid content was determined by aluminium chloride colorimetric technique as described previously [16].

### Determination of 1,1-diphenyl-2-picrylhydrazyl antioxidant activity (DPPH)

The radical scavenging activity was measured according to an assay described previously with modifications [15]. Briefly, diluted samples (50μL, 0.5–5 mg/mL) were added with DPPH (150μL, 0.2 mM) and incubated for 30 min in the dark. After incubation the absorbance was measured at 490 nm. Butyl hydroxyl toluene (BHT, 500μg/mL) was used as a positive control. DPPH activity was expressed as percent inhibition (%) = ((A_control_ – A_sample_)/A_control_ × 100).

### Determination of ferric reducing antioxidant power (FRAP)

The reducing power of the samples was determined as described previously [17]. Ascorbic acid (0-250μg/mL) was used to develop a standard curve and results calculated and expressed as ascorbic acid equivalents (μg AAE/g).

### Statistical analysis

Results were expressed as the mean ± standard deviation (SD) or standard error of the mean (SEM) from three independent experiments. IC_50_ values were calculated by non-linear regression using Graphpad Prism Version 6.0 for Windows. One-way analysis of variance (ANOVA) was determined using Tukey’s or Dunnet’s multiple comparison tests and linear relationship was determined with Pearson’s correlation coefficient (r^2^). Difference were considered to be significant at *p* < 0.05.

## Results and discussion

Natural products containing polyphenols and antioxidants may be an effective way of reducing oxidative stress and delaying the onset of diabetic complications. Dietary or nutritional supplements are commonly used for general wellness and prevention of disease. Nutritional supplements including herbal extracts and fruit juices may contain pharmacoactive components, and whilst manufacturers claim that these components provide health benefits, very little scientific evidence is available to-date to support these claims. In this study we aimed to investigate the antiglycation, α-amylase and α-glucosidase inhibitory potential of selected liquid nutritional supplements.

### α-amylase and α-glucosidase inhibition

**Fig. 1:**
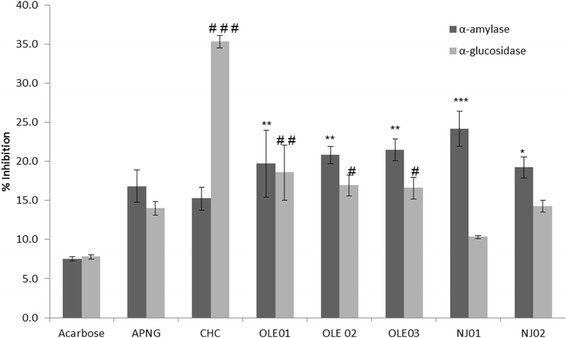
The percentage inhibition of liquid dietary supplements on α-amylase and α-glucosidase activity. Values are expressed as mean ± SEM, *n* = 3. *p < 0.05, **p < 0.01, ***p < 0.001 are significant different when compared to α-amylase values for acarbose. #*p* < 0.05, ## *p* < 0.01, ###*p* < 0.001 are significantly different when compared to α-glucosidase values for acarbose. Supplements include acai powder (APNG), chlorophyll (CHC), olive leaf extracts (OLE01, OLE02 OLE03) and noni juice (NJ01, NJ02)

One of the primary targets for the management of early stage T2DM is to maintain steady blood glucose levels and prevent the postprandial surge of glucose, and an effective way of achieving this is by inhibiting α-amylase or α-glucosidase [7]. In our study, α-amylase and α-glucosidase inhibitory potential of liquid nutritional supplements were compared with a positive control (acarbose). Inhibition of α-amylase ranged from 15.22 ± 1.46 to 24.14 ± 2.23 % (Fig. 1). Samoan noni juice (NJ01) showed moderate but significant α-amylase inhibition (*p* < 0.001) whereas APNG and CHC showed no difference when compared to acarbose. Olive leaf supplements (OLE01, OLE02 and OLE 03) showed similar level of inhibition and were significantly higher (*p* < 0.01) than acarbose. Our study supports the findings of Komaki and co-workers [18] who found ethanolic extracts of olive leaf to effectively inhibit α-amylase and determined oleuropein to be one of the main phenolic compounds responsible for this inhibitory effect. In our study, all olive leaf supplements contained 4.4 mg/mL of oleuropein (determined on the product label). A previous study using noni juice when taken twice a day for 20 days also demonstrated hypoglycaemic effects in diabetic rats but α-amylase levels were not measured [19].

Inhibition of α-glucosidase ranged from 10.33 ± 0.25 to 35.29 ± 0.93 %, with CHC showing the highest inhibition (Fig. 1). All olive leaf supplements (OLE01, OLE02 and OLE03) demonstrated inhibitory effects that were significantly greater than acarbose. APNG, NJ01 and NJ02 exhibited inhibitory activity at levels similar to that of acarbose. Previous reports have shown the use olive leaf extracts for improving diabetic complications [20, 21], however, this is the first study to report on α-glucosidase inhibitory activity by chlorophyll and olive leaf extracts. Previous studies investigated various plant and fruit extracts for the inhibition of α-glucosidase and attributed their potential inhibitory activities to anthocyanin content [12, 22]. In our study, CHC extract contained chlorophyll including anthocyanin, ascorbic acid and potassium sorbate, which may have played a role in its inhibitory effects. Isolation of key components from CHC and their enzyme inhibitory potentials needs further investigation. Another limitation in our study was that the inhibitory potential against both enzymes were investigated only at one concentration thus a concentration dependent study would allow comparisons with other studies and known inhibitors.

### Antiglycation

The use of pharmacological compounds that could inhibit the formation of AGEs is another approach in delaying or preventing the onset of diabetic complications. Aminoguanidine, a well investigated inhibitor of protein glycation prevents AGE formation by reacting with carbonyl groups on reducing sugars [3] however this clinical inhibitor has numerous side effects [23]. In our study, the inhibitory potential of liquid nutritional supplements on fluorescent protein-bound AGEs was explored using BSA-glucose models. IC_50_ values ranged from 0.5 ± 0.03 to 4.70 ± 0.67 mg/mL (Table 1).

**Table 1:** Total phenolic content, total flavonoid content, ferric reducing antioxidant potential and antiglycation activities of liquid nutritional supplements

	TPC (mgGAE/mL)	TFC (mgQE/mL)	FRAP (mgAAE/mL)	Fluorescent AGE (IC_50_, mg/mL)
APNG	1.77 ± 0.12^b^	0.86 ± 0.35^b^	2.52 ± 0.16^c^	2.84 ± 1.01^c^
CHC	2.35 ± 0.09^c^	1.15 ± 0.29^c^	3.16 ± 0.14^d^	1.60 ± 0.57^bc^
OLE01	1.98 ± 0.06^bc^	1.03 ± 0.21^b^	2.01 ± 0.25^b^	2.14 ± 0.21^bc^
OLE02	2.65 ± 0.13^d^	1.52 ± 0.12^c^	1.70 ± 0.06^b^	1.03 ± 0.10^ab^
OLE03	3.00 ± 0.36^e^	1.57 ± 0.97^c^	1.95 ± 0.17^b^	0.50 ± 0.03^a^
NJ01	0.48 ± 0.03^a^	0.09 ± 0.00^a^	0.73 ± 0.06^a^	4.70 ± 0.67^d^
NJ02	0.05 ± 0.02^a^	0.06 ± 0.03^a^	0.94 ± 0.08^a^	1.89 ± 0.43^bc^

Data values are expressed as mean ± SD, *n* = 3. Different superscripts indicate significant difference (*p* < 0.05) within each column. Supplements include acai powder (APNG), chlorophyll (CHC), olive leaf extracts (OLE01, OLE02 OLE03) and noni juice (NJ01, NJ02)

OLE03 showed to be the most effective inhibitor with the lowest IC_50._ These results support the findings of a previous study which found methanolic olive leaf extract to be a successful inhibitor of protein glycation in BSA-ribose models (3 weeks incubation), inhibiting fluorescent AGE formation [24]. The authors suggested that luteolin, luteolin-4’-O-β- D-glucopyranoside, oleuropein and hydroxytyrosol may be responsible for the inhibitory effect. In our study, olive leaf supplement (OLE03) sold as blood sugar support supplement contained oleuropein (4.4 mg/mL) and hydroxytyrosol (250 μg/mL) that may have contributed to lower AGE formation.

### Correlations between inhibitory activities and phenolic or flavonoid or antioxidant activities

**Fig. 2:**
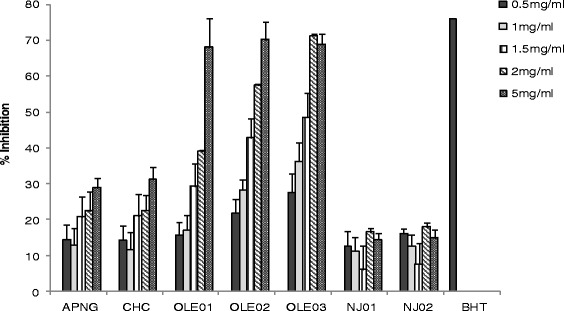
The percentage inhibition of liquid dietary supplements on 1,1-diphenyl-2-picrylhydrazyl antioxidant activity (DPPH). Values are expressed as mean ± SD, *n* = 3. Samples include acai powder (APNG), chlorophyll (CHC), olive leaf extracts (OLE01, OLE02 OLE03), noni juice (NJ01, NJ02) and Butyl hydroxyl toluene (BHT)

TPC ranged from 0.48 ± 0.03 to 3.00 ± 0.36 mg GAE/ mL whereas TFC ranged from 0.06 ± 0.03 to 1.52 ± 0.97 mg QE/mL in liquid nutritional supplement (Table 1). The free radical scavenging activities of liquid nutritional supplements showed a dose dependent response (Fig. 2). FRAP values ranged from 0.73 ± 0.06 to 3.16 ± 0.14 mg AAE/mL of sample (Table 1). Pearson’s correlation analysis detected several significant relationships among the investigated variables. Correlation analysis showed DPPH free radical scavenging activity to be positively correlated with TPC (r^2^ = 0.6329, *p* = 0.0021) but not with TFC. Interestingly, FRAP results showed a strong positive correlation with TPC (r^2^ = 0.647, *p* < 0.001) and TFC (r^2^ = 0.840, *p* < 0.0001). These relationships show that the total phenolic content defines their antioxidant activities.

The α-amylase inhibition did not correlate with TPC, TFC or antioxidant activities however, α-glucosidase inhibition positively correlated with TFC (r^2^ = 0.766, *p* < 0.01) and FRAP (r^2^ = 0.750, *p* < 0.01) (Table 2). There was no significant correlation between α-glucosidase inhibition and TPC or DPPH scavenging activity (Table 2).

**Table 2:** Pearson’s correlation coefficient between different variables

	TPC	TFC	FRAP	DPPH
Antiglycation (IC_50_)	−0.707***	ns	ns	−0.515*
α-amylase inhibition	ns	ns	ns	ns
α-glucosidase inhibition	ns	0.766**	0.750**	ns

*p < 0.05,**p < 0.01, **p < 0.001, *ns* correlation not significant

Various levels of correlation between TPC, TFC and antioxidant activities and enzyme inhibition are reported in the literature. For example, no linear relationship was identified between α-glucosidase inhibitory activities of native Australian herb fractions and their FRAP values [25]. A moderate positive relationship between TPC and α-glucosidase inhibition for commonly used medicinal plants (r^2^ = 0.393) and herbal teas (r^2^ = 0.371) has been reported [26]. In the present study, the result shows different affinities of evaluated variables to enzyme inhibition that relates to different relationships reported in the literature, suggesting that the total phenolic, flavonoids and their antioxidant capacities do not always define enzyme inhibitory activities. IC_50_ values for fluorescent AGE inhibitions negatively correlated with TPC (r^2^ = −0.707, *p* < 0.001) and DPPH scavenging activity (r^2^ = −0.515, *p* < 0.05). Positive relationships were observed with wild berries, where % AGE inhibition correlated with TPC (r^2^ = 0.760) and DPPH (r^2^ = 0.452) [5]. Antiglycation agents may exert their effects by blocking free amino groups on proteins, or carbonyl groups on reducing sugars so that they cannot bind; acting as antibodies to block Amadori products; as enzymes deglycating Amadori products and intermediates; and chelating transition metals, reducing glycation-derived free radicals [3]. In our study antiglycation activity correlated with TPC and DPPH, suggesting that phenolic compounds may protect against glycation; preventing the conversion of Amadori products into AGEs that occurs in the presence of transition metals and oxygen.

## Conclusion

Commercial nutritional supplements analysed in this study exhibited different levels of inhibitory activity against α-amylase, α-glucosidase and protein-bound AGEs. While some supplements were more effective than others these inhibitory activities did not always correlate with phenolic content, flavonoid or antioxidant activities, suggesting that the inhibition may occur via different pathways. This study highlights promising potential of liquid nutritional supplements as a therapeutic approach to managing T2DM. As this study was the first to investigate antiglycation potential of liquid nutritional supplements, it provides opportunity to explore more neutraceutical supplements and their relevant metabolites in cells, animal or humans to determine the bioavailability and the mechanism of action.

## Abbreviations

AGEs: Advanced glycation endproducts
APNG: Acai power, Acai juice blend
CHC: Chlorophyll, Detox health drink
DPPH: 1,1-diphenyl-2-picrylhydrazyl antioxidant activity
FRAP: Ferric reducing antioxidant power
NJ01: Noni Juice, Samoan
NJ02: Fijian Noni
OLE01: Olive leaf extract, Natural High Strength
OLE02: Olive leaf extract, High Strength
OLE03: Olive Leaf Extract, Blood Sugar Support
ROS: Reactive oxygen species
T2DM: Type 2 diabetes mellitus
TFC: Total flavonoid content
TPC: Total phenolic content
